# Enhancing competency and self-directed learning in anesthesiology residency: an outcome-based education model integrating online–offline hybrid teaching and mind mapping: a randomized controlled trial

**DOI:** 10.3389/fmed.2025.1684116

**Published:** 2026-01-09

**Authors:** Rili Yu, Cancan Cheng, Fan Zhang

**Affiliations:** Department of Anesthesiology, The Third Xiangya Hospital, Central South University, Changsha, Hunan, China

**Keywords:** outcome-based education, online–offline hybrid teaching, mind mapping, anesthesiology, standardized residency training, teaching effectiveness

## Abstract

**Objective:**

While outcomes-based education (OBE) has been widely advocated for decades, its intentional integration with a hybrid (online–offline) instructional model in residency training remains underexplored. This study aimed to evaluate the effectiveness of an integrated OBE approach combined with an online–offline hybrid teaching model and mind mapping in the standardized training of anesthesiology residents.

**Methods:**

Sixty anesthesiology residents undergoing standardized training were randomly divided into a control group and a study group (*n* = 30 each). The control group received traditional didactic teaching, while the study group received OBE-guided instruction using a blended online–offline model supplemented by mind mapping strategies. The two groups were compared in terms of baseline characteristics, theoretical knowledge, practical skill scores, teaching satisfaction, instructor evaluations, and self-directed learning abilities.

**Results:**

The study group achieved significantly higher scores in both theoretical and practical assessments compared with the control group, and independent-samples *t*-tests indicated that the differences were statistically significant (*t* = −5.451, −6.551, *p* < 0.05). Trainees in the study group reported greater satisfaction with the teaching model compared with the control group, particularly in teamwork, teacher–student communication, learning enthusiasm, clinical applicability, clinical thinking ability, and overall teaching satisfaction. Chi-square tests showed statistically significant differences between the two groups (χ^2^ = 5.455, 7.680, 5.192, 9.231, 4.320, 6.667; *p* = 0.020, 0.006, 0.023, 0.002, 0.038, 0.010). Instructors’ evaluations of the study group residents were also higher than those of the control group in basic theoretical knowledge, learning attitude, problem-solving ability, and communication skills. The excellence rates for these aspects were significantly greater in the study group (χ^2^ = 5.962, 6.667, 5.455, 5.455; *p* = 0.015, 0.010, 0.020, 0.020). In addition, the study group performed significantly better than the control group across all dimensions and in the total score of self-directed learning ability (*t* = −13.163, *p* < 0.001).

**Conclusion:**

Incorporating outcome-based education with an online–offline hybrid teaching model and mind mapping effectively enhances the academic performance, clinical competence, and self-directed learning capabilities of anesthesiology residents. This comprehensive instructional approach holds strong potential for broader adoption in standardized medical education and residency training programs.

## Introduction

Outcome-based education (OBE), first conceptualized several decades ago, represents a well-established paradigm in medical education paradigms, emphasizing pre-defined goals and measurable outcomes rather than traditional process-focused approaches. As defined by Spady, one of its leading advocates, OBE focuses on designing and developing instruction where goals and outcomes are clearly established before curriculum development (1). Over the past decades, this educational model has significantly influenced by centering decision-making on learners’ competence at crucial educational milestones, enabling criterion-referenced evaluation and promoting individualized, standard-oriented education that enhances overall educational quality (2). During the COVID-19 pandemic, online and blended (hybrid) learning rapidly became essential modes of instruction worldwide. Dedeilia et al. (3) provided a comprehensive analysis of this transformation, reporting that learners were highly satisfied with these formats and favored their ongoing implementation beyond the pandemic period. This evidence supports the sustainability and effectiveness of hybrid educational models in medical training. In this context, hybrid online–offline learning has evolved into a sustainable and mature educational model that combines the advantages of digital and traditional learning environments (4). The hybrid teaching method strategically combines traditional face-to-face instruction with online components, creating a comprehensive learning environment that maximizes the benefits of both modalities (5). This integration requires careful consideration of platform architecture, component interventions, and specific educational materials to ensure effective knowledge transfer and skill development.

Building upon the development of hybrid learning, the flipped classroom model has also gained increasing recognition as an effective learner-centered approach in medical education. In this model, students review learning materials prior to class and engage in active discussions and problem-solving during classroom sessions. This design promotes self-directed learning and interactive engagement, complementing both outcome-based and blended learning frameworks to further enhance the efficiency and interactivity of medical training (6, 7). Mind mapping, innovatively developed by Tony Buzan, has emerged as a powerful visual learning tool in medical education. This technique creates non-linear representations of ideas linked to a central core topic, engaging both hemispheres of the brain through a multi-sensory approach incorporating words, concepts, colors, and symbols (8). The unique combination of visual elements and structured organization in mind maps not only facilitates memory retention but also accommodates diverse learning styles, making it particularly effective in medical education where complex interrelationships between concepts must be understood and retained (9).

In the specific context of anesthesiology education, traditional training approaches have shown limitations in providing consistent and comprehensive skill development. While structured curricula have demonstrated improvements in learner satisfaction and clinical management skills, the field faces challenges in standardizing training experiences and ensuring uniform competency development (10). Current training systems, particularly in residency programs, require enhancement to address emerging challenges in clinical practice and public health emergencies (11). The implementation of structured curricula incorporating case-based learning, problem-based discussion, and simulation has become essential for improving both technical and non-technical skills in anesthesiology training (12). The integration of competency-based curricula in medical education aims to develop residents who can perform effectively within established standards of practice. This approach emphasizes the building of knowledge and skills in a progressive, interconnected manner (13). Systematic evaluations have demonstrated that blended learning formats can significantly enhance clinical skills and reflective capabilities among medical students, while incorporating self-directed learning tools such as video recording and self-annotation has shown promising results in improving learning outcomes (14).

While both OBE and hybrid learning have been extensively implemented in medical education, their intentional integration—combined with flipped classroom design and mind mapping techniques—has rarely been systematically explored in anesthesiology residency training. This study was therefore designed to investigate the comprehensive effects of combining OBE principles with online–offline blended teaching, flipped classroom design, and mind mapping techniques in the standardized training of anesthesiology residents.

## Materials and methods

### Ethical approval

All experimental procedures were approved by the Medical Ethics Committee of The Third Xiangya Hospital, Central South University in accordance with Helsinki Declaration. All participants signed informed consent forms.

### Sample size estimation and participant selection

The sample size was estimated based on statistical power analysis using G*Power 3.1.9.7 software (Heinrich Heine University, Düsseldorf, Germany). With a power of 0.80 (in this study, the effect size was set at 0.8 (indicative of a “large effect”) for the primary outcome of anesthesiology residents’ theoretical examination scores. This specification was based on evidence from comparable educational intervention studies (15, 16)), a significance level (*α*) of 0.05, an effect size of 0.8, and two comparison groups, the minimum required sample size was calculated to be 52 participants. Considering a potential 10% attrition rate, the final target sample size was rounded up to 60 resident physicians. A total of 60 anesthesiology residents undergoing standardized residency training in the Department of Anesthesiology at our hospital between June 2023 and June 2024 were initially recruited. Randomization was performed using the random number table method. A dedicated statistician generated random numbers from 1 to 60 using SPSS Statistics 25.0 (IBM Corp., Chicago, IL, USA). Eligible participants were numbered sequentially and randomly assigned in a 1:1 ratio to either the control group or the intervention group. The control group received traditional teaching, while the intervention group received online–offline blended teaching based on OBE principles (Details are provided in the Methods section or Table 1). Blinding was not applied in this study.

**Table 1:** Comparison of teaching methods between the control and study groups.

Teaching dimension	Control group (traditional residency teaching)	Study group (mind mapping + OBE blended online–offline teaching)
Core concept	Follows the residency training manual, teacher-centered instruction	Centered on OBE, focusing on competency cultivation and building a closed-loop teaching process
Teaching format	Fully offline instruction	Blended online and offline teaching divided into three stages: pre-class, in-class, and post-class
Core tools	Multimedia equipment	Multimedia equipment + XMind mind mapping software
Frequency and duration of sessions	Twice per week, 2 h per session, 30 min of practical guidance per session	Twice per week, 2 h per session, 30 min of practical guidance per session
Key teaching components	1. Case introduction; 2. Teacher-led theoretical and practical instruction; 3. Student summary and test; 4. Post-class self-review	1. Pre-class: Online distribution of preview materials, mind mapping training, and submission of preview maps; 2. In-class: Layered instruction assisted by mind maps, case discussion, and optimization of thinking through map comparison; 3. Post-class: Refinement of mind maps, online testing, and sharing of excellent maps
Interaction method	Classroom questioning and student summaries	Real-time online interaction before class, case discussions and presentations during class, and instructor feedback and corrections after class

### Educational background

All participants were graduates of Chinese medical universities majoring in clinical medicine and had passed the National Medical Licensing Examination. According to the national standards for standardized residency training in China, the duration of anesthesiology residency is 3 years. There were no significant differences between the two groups in terms of training year or educational level (*p* > 0.05; Table 2), indicating good comparability. All residents received instruction in Chinese at the Anesthesiology Residency Teaching Center of our hospital, where the teaching facilities and hardware conditions were consistent. Both groups received lessons lasting 2 h per session, twice per week, delivered by the same team of attending physicians with more than 5 years of teaching experience who prepared lessons jointly to ensure instructional consistency. During the study period, the control group was not exposed to any of the intervention materials; however, to ensure educational equity, the new teaching approach was provided to them 1 month after the completion of the study.

**Table 2:** Comparison of general information between the control and study groups.

Information	Control group (*n* = 30)	Study group (*n* = 30)	χ^***2***^*/t*	*P*
Age (years)	26.20 ± 1.19	26.50 ± 0.97	−1.071	0.289
Gender			0.606	0.436
Male	12 (40%)	15 (50%)		
Female	18 (60%)	15 (50%)		
Educational background			1.121	0.571
Bachelor degree	7 (23%)	9 (30%)		
Master degree	19 (63%)	15 (50%)		
Doctoral degree	4 (13%)	6 (20%)		
Prior experience in mind mapping	6 (20%)	4 (13%)	0.480	0.488
Duration of standardized training			0.650	0.722
First year	9 (30%)	11 (37%)		
Second year	8 (27%)	9 (30%)		
Third year	13 (43%)	10 (33%)		

### Inclusion criteria

(1) Residents specializing in anesthesiology undergoing standardized training at our hospital; (2) Voluntary participation in this study.

### Exclusion criteria

(1) Residents rotating through the anesthesiology department who are not specializing in anesthesiology; (2) Residents who withdrew from the training program midway; (3) Residents with poor cooperation and low motivation, defined as two consecutive unexcused absences from scheduled in-person teaching sessions.

### Control group

The control group received traditional teaching methods in accordance with the standardized residency training manual. After joining the department, the residents participated in clinical activities such as medical record writing. Following the established teaching syllabus and integrating clinical practice, the instructors conducted classroom teaching primarily through multimedia presentations and face-to-face lectures (offline group sessions, 2 h per session, twice per week). The instructors explained the fundamental theories and practical skills of clinical anesthesia, demonstrated key procedures (followed by one-on-one corrective guidance lasting approximately 30 min per session), and adopted a teacher-centered, lecture-based approach. Clinical cases were introduced at the beginning of each class to contextualize the lesson, followed by systematic explanations of the relevant theoretical content. The instructors encouraged critical thinking and posed guiding questions to the trainees. After each class, residents were required to summarize the key points and complete assessments identical to those used in the study group. The instructors also advised them to conduct independent review and consult supplementary materials after class.

### Study group

The study group implemented a teaching method that combines mind mapping with OBE principles, integrating both online and offline instruction.

Pre-class (online teaching component): The preview PowerPoint slides for the upcoming anesthesiology residency training session—covering the core theoretical framework of the next topic—were shared in the class WeChat group along with a pre-learning guide. The guide outlined the key knowledge points to be reviewed, relevant foundational medical content, and an introduction to mind mapping, including its purpose, the use of XMind software, and techniques for creating mind maps. Each student was required to create a personal mind map to ensure proficiency in software use and familiarity with the topic. Students accessed and downloaded the learning materials independently and interacted with the instructor on the platform whenever questions arose during self-study. The instructor assessed students’ preparation by reviewing their questions and feedback submitted online.

**Figure 1:**
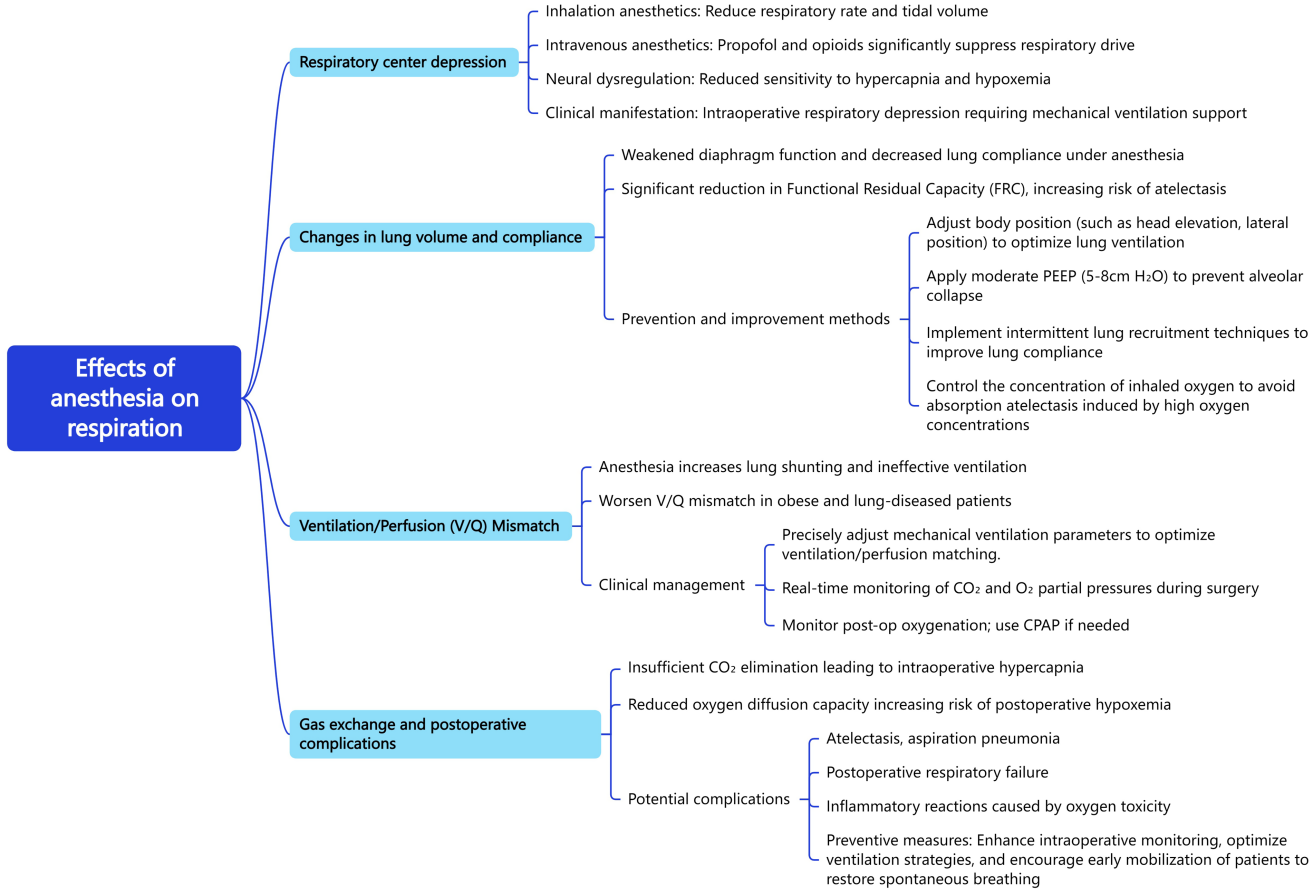
Example of a mind map for clinical teaching on the effects of anesthesia on respiration.

In-class (offline teaching component): During classroom sessions, the instructor used self-developed mind maps to organize and explain key concepts systematically and to link related knowledge points, ultimately summarizing them into an integrated knowledge framework. Based on students’ online progress, offline clinical mini-lectures and case discussions were held (2 h per session, twice weekly). This process reinforced memory, comprehension, and clinical application of theoretical knowledge. Students were encouraged to participate actively in classroom discussions and compare their own mind maps with the instructor’s version—for example, when discussing the impact of anesthesia on respiration (Figure 1). Using the mind mapping method, students listed primary and secondary keywords such as “respiratory center depression,” “changes in lung volume and compliance,” “ventilation/perfusion mismatch (V/Q ratio),” and “gas exchange and postoperative complications.” This activity fostered divergent thinking and helped students develop clear, structured summaries that enhanced both theoretical understanding and clinical reasoning. Immediate corrective guidance lasting approximately 30 min followed each procedural demonstration.

Post-class (online teaching component): After class, students refined and supplemented their pre-class mind maps by incorporating new knowledge learned during the session. Post-class online tests were administered once a week for a total of 12 sessions. Each test contained 25 questions: 15 single-choice items (4 points each) and 10 multiple-choice items (4 points each), for a total score of 100 points. The time limit was set at 25 min. Instructors analyzed the results to provide targeted feedback in subsequent sessions. Each group further optimized its mind map, ensuring logical structure, clear hierarchy, focused content, and visually coherent color schemes. Exemplary mind maps were uploaded by the instructor to the online platform for peer review and collective learning.

Both groups were taught by the same residency teaching team (*n* = 5), all of whom were attending anesthesiologists or above with ≥ 5 years of teaching experience. The team conducted unified lesson preparation and standardized the teaching requirements. The two groups received identical teaching duration and frequency (both groups followed a 12-week teaching cycle, for a total of 24 teaching hours); only the instructional method differed, thereby ensuring that the intervention constituted the sole between-group difference.

### Outcomes and measures

Primary outcome: Upon completion of the course, theoretical and practical assessments in anesthesiology were conducted. Theoretical assessment: This examination evaluated residents’ knowledge retention and understanding of anesthesiology concepts. The test comprised multiple-choice questions (30 points, 30 questions, 1 point each), fill-in-the-blank questions (40 points, 20 questions, 2 points each), and case analysis/problem-solving questions (30 points, 2 questions, 15 points each). The total score was 100 points, accounting for 60% of the overall grade. The examination was administered as a closed-book online test using the Chaoxing Learning Platform. The implementation details were as follows: ① Examination platform: Chaoxing Learning Platform served as the core testing system. ② Implementation method: A unified examination time (e.g., Saturday 9:00–10:00) was established. The system automatically distributed the test papers, with a two-hour countdown timer. Upon completion, the system auto-submitted responses. Objective questions were automatically graded by the system, while subjective case analysis questions were independently scored by two senior instructors in a double-blind manner. When the scoring difference was ≤ 5 points, the average was taken; if the difference exceeded 5 points, a third instructor conducted a review. ③ Proctoring measures: The system incorporated facial recognition for pre-exam identity verification and screen-switching detection (automatic submission triggered after more than three unauthorized screen switches). Practical assessment: The practical examination assessed residents’ procedural performance and included evaluation of detection and assessment skills (30 points), the “three-party verification” process (30 points), and operational skills (40 points), for a total of 100 points, accounting for 40% of the overall grade. Two chief anesthesiologists who were not involved in daily teaching independently graded the performance using a unified scoring rubric, and the mean of their scores was recorded as the final result.

Secondary outcomes:

1) Residents’ feedback on teaching satisfaction and perceptions: After course completion, a questionnaire survey was conducted among both groups to collect feedback from anesthesiology residents on the two teaching models. The questionnaire assessed the following aspects: enhancement of teamwork, improvement of teacher–student communication, stimulation of learning enthusiasm, increase in clinical applicability, development of clinical thinking ability, and overall teaching satisfaction. A five-point Likert scale was used (1 = very dissatisfied, 2 = dissatisfied, 3 = neutral, 4 = satisfied, 5 = very satisfied). Scores of 4 (“satisfied”) and 5 (“very satisfied”) were combined and defined as “satisfied,” while scores of 1–3 were combined and defined as “not satisfied.” A total of 60 questionnaires were distributed and 60 valid responses were collected, yielding a 100% valid response rate.
2) Instructors’ evaluation of residents’ learning performance: After the course, feedback from anesthesiology teaching instructors was obtained through a structured questionnaire designed according to previously published literature. The questionnaire covered four domains: basic theoretical knowledge, learning attitude, problem-solving ability, and communication skills. Each item was rated using a five-point Likert scale (1 = very poor, 2 = poor, 3 = fair, 4 = good, 5 = excellent). Scores of 4 and 5 were categorized as “good or excellent,” while scores of 1–3 were defined as “below good.” The survey was conducted in person after the weekly departmental meeting. The researcher distributed printed questionnaires to instructors on site, explained the completion instructions, and collected them immediately. Returned questionnaires were checked one by one, and those with ≥ 2 missing responses or logical inconsistencies (e.g., multiple selections within a single dimension) were excluded as invalid. A total of five instructors participated in the evaluation, all of whom were attending anesthesiologists or above with at least 5 years of residency teaching experience. Sixty questionnaires were distributed and all 60 were validly returned (effective recovery rate = 100%).
3) Self-directed learning ability: Following course completion, the Chinese version of the Self-Directed Learning Ability Scale developed by Shen Wangqin (17) was administered to both groups. The scale consists of five dimensions—learning awareness, learning behavior, learning strategies, learning evaluation, and interpersonal skills—with 12 items in each dimension, totaling 60 items. The scale has demonstrated high reliability and validity (Cronbach’s *α* = 0.966, test–retest reliability = 0.855, content validity = 0.963). A five-point Likert scale was used (5 = always, 4 = often, 3 = sometimes, 2 = rarely, 1 = never), and all items were positively scored. The total score was calculated as the sum of all 60 items (range: 60–300 points), with higher scores indicating stronger self-directed learning ability. The questionnaire was administered online: researchers distributed a unified link to both groups via the designated platform, provided clear instructions to ensure honest and objective responses, and enabled access for completion. Upon submission, responses were automatically collected by the system. A total of 60 questionnaires were distributed and 60 valid responses were recovered (effective response rate = 100%) (18).

### Statistical analysis

All data were analyzed using SPSS 26.0 statistical software. Continuous data were expressed as mean ± standard deviation (Mean ± SD) and analyzed using *t*-tests. Categorical data were presented as [n]% and analyzed using chi-square tests. If any expected frequency was less than 5, Fisher’s exact test was employed. A *p*-value < 0.05 was considered statistically significant.

## Results

### General information

A total of 70 resident physicians were recruited. Among them, 4 residents did not meet the inclusion criteria, and 6 were excluded according to the exclusion criteria (including 4 residents from non-anesthesiology specialties rotating in the department and 2 with poor compliance), resulting in 60 eligible participants.

The comparison of general characteristics between the study group and the control group of resident physicians showed no statistically significant differences in age, gender, educational background, prior experience in mind mapping, or years of residency training, as determined by independent-samples t-tests and chi-square tests (t/χ^2^ = −1.071, 0.606, 1.121, 0.480, 0.650; *p* = 0.289, 0.436, 0.571, 0.488, 0.722) (*p* > 0.05), indicating comparable baseline characteristics between the two groups (Table 2).

### Theoretical and practical examination scores, and self-directed learning abilities

The comparison of theoretical and practical examination scores between the two groups of standardized training physicians revealed that the study group achieved significantly higher total scores in both the theoretical and practical examinations than the control group (*t* = −5.451, −6.551; *p* < 0.001). These results indicate that the study group outperformed the control group in both theoretical learning and practical operation (Table 3). The comparison of self-directed learning abilities between the two groups of resident physicians revealed that the study group scored significantly higher than the control group across all dimensions—awareness, strategies, behaviors, evaluation, and interpersonal skills—as well as on the total score. Independent-samples t-tests indicated statistical significance for all comparisons (t = −4.207, −7.754, −3.472, −6.499, −5.223, −13.163; all *p* < 0.001). These results indicate that the study group residents demonstrated superior self-directed learning ability across all domains (Table 3).

**Table 3:** Theoretical and practical assessment scores and self-directed learning ability.

	Control group (*n* = 30)	Study group (*n* = 30)	*t*	*P*
Assessment scores				
Multiple choice	22.40 ± 1.99	24.30 ± 2.45	3.294	0.002
Fill-in-the-Blank	30.40 ± 2.71	31.87 ± 2.73	2.089	0.041
Case analysis	20.50 ± 4.50	25.10 ± 2.77	4.767	<0.001
Total theoretical score	73.30 ± 6.23	81.27 ± 5.03	−5.451	<0.001
Assessment evaluation	22.30 ± 2.77	24.90 ± 3.45	3.221	0.002
Tri-party verification	24.07 ± 2.95	26.93 ± 4.08	3.122	0.003
Skill operation	29.93 ± 4.26	32.37 ± 4.13	2.246	0.029
Total practical score	76.30 ± 4.82	84.20 ± 4.51	−6.551	<0.001
Self-directed learning ability				
Learning awareness	41.97 ± 3.34	46.10 ± 4.22	−4.207	<0.001
Learning strategies	43.07 ± 2.70	48.10 ± 2.31	−7.754	<0.001
Learning behaviors	39.97 ± 2.76	43.23 ± 4.35	−3.472	0.001
Learning evaluation	41.30 ± 2.83	46.70 ± 3.56	−6.499	<0.001
Interpersonal skills	41.33 ± 4.30	46.83 ± 3.84	−5.223	<0.001
Total score	207.63 ± 6.01	230.97 ± 7.62	−13.163	<0.001

### Feedback from resident physicians and instructors

The comparison of feedback from resident physicians in both groups regarding the two teaching models revealed that the satisfaction rates of the study group were higher than those of the control group in the aspects of teamwork enhancement, teacher-student communication improvement, learning enthusiasm stimulation, clinical practicality enhancement, clinical thinking skills cultivation, and teaching satisfaction. According to the chi-square test (χ^2^ = 5.455, 7.680, 5.192, 9.231, 4.320, 6.667; *p* = 0.020, 0.006, 0.023, 0.002, 0.038, 0.010), the differences were statistically significant. These results indicate that the teaching model adopted by the study group better meets the needs of resident physicians, particularly in areas such as teamwork, teacher-student communication, clinical practicality, and the cultivation of thinking skills (Table 4).

**Table 4:** Comparison of feedback from resident physicians and instructors on the learning outcomes of residents between the control and study groups.

Residents’ evaluation of the teaching model	Control group (*n* = 30)		Study group (*n* = 30)		*χ^2^*	*P*
	Satisfied	Dissatisfied	Satisfied	Dissatisfied		
Teamwork enhancement	21 (70%)	9 (30%)	28 (93%)	2 (7**%**)	5.455	0.020
Teacher-student communication improvement	21 (70%)	9 (30%)	29 (97%)	1 (3%)	7.680	0.006
Learning enthusiasm stimulation	23 (77%)	7 (23%)	29 (97%)	1 (3%)	5.192	0.023
Clinical practicality enhancement	22 (73%)	8 (27%)	30 (100%)	0 (0%)	9.231	0.002
Clinical thinking skills cultivation	22 (73%)	8 (27%)	28 (93%)	2 (7%)	4.320	0.038
Teaching satisfaction	20 (67%)	10 (33%)	28 (93%)	2 (7%)	6.667	0.010

Instructors’ feedback on residents’ learning performance	Control group (*n* = 30)		Study group (*n* = 30)		*χ^2^*	*P*
	Excellent	Not excellent	Excellent	Not excellent		
Basic theoretical knowledge	19 (63%)	11 (37%)	27 (90%)	3 (10%)	5.962	0.015
Learning attitude	20 (67%)	10 (33%)	28 (93%)	2 (7%)	6.667	0.010
Problem-solving ability	18 (60%)	12 (40%)	25 (83%)	5 (17%)	5.455	0.020
Communication skills	21 (70%)	9 (30%)	28 (93%)	2 (7)%	5.455	0.020

The feedback from instructors regarding the learning outcomes of the two groups of resident physicians revealed that the rate of excellent or good evaluation in the study group were markedly higher than those in the control group for students’ foundational theoretical knowledge, learning attitude, problem-solving ability, and communication skills. Chi-square tests indicated statistically significant differences (χ^2^ = 5.962, 6.667, 5.455, 5.455; *p* = 0.015, 0.010, 0.020, 0.020). These results indicate that the excellent rates for the study group were significantly higher than those for the control group across all evaluation metrics (Table 4).

## Discussion

In the medical training field, educators are challenged to develop curriculum structures that are coherent, coordinated, and integrated within and across different educational interventions (13). This study evaluated a novel teaching approach that integrates OBE, hybrid online–offline learning, and mind mapping within anesthesiology residency training. This integration represents a pedagogical innovation aimed at bridging theoretical and practical competencies, promoting self-directed learning, and enhancing clinical decision-making.

From the perspective of adult learning theory proposed by Malcolm Knowles, adult learners are self-directed, problem-oriented, and internally motivated to apply learning to real-life contexts. In medicine, residency education represents a quintessential example of adult learning, where trainees are autonomous professionals who benefit from self-directed and experience-based learning environments. The blended OBE–mind mapping model developed in this study aligns with Knowles’ andragogical principles by empowering residents to take ownership of their learning process, set personal learning goals, and integrate knowledge through reflective practice. This approach is consistent with current evidence emphasizing that self-directed, experiential learning enhances physicians’ competence, motivation, and long-term professional development (19). Recent evidence has also confirmed that learner autonomy and perceived relevance—core components of adult learning—are key predictors of engagement and satisfaction in residency training (20). The results align with existing literature in several key aspects. First, the significant improvement in test scores mirrors previous findings where mind mapping significantly enhanced both short-term and long-term memory retention in medical education, particularly in technical subjects (8).

From a cognitivist standpoint, learning involves the active organization and restructuring of information into meaningful cognitive schemas. Mind mapping facilitates this process by externalizing mental representations of complex systems and promoting deeper processing of interrelated concepts. Recent cognitive load research in medical education has demonstrated that structured visual tools can reduce extraneous cognitive load, freeing mental resources for higher-order reasoning and problem-solving (21). This mechanism helps explain why mind mapping effectively supports knowledge retention and transfer. Sajadi et al. confirmed that medical students using mind mapping exhibited superior long-term recall and clinical reasoning scores compared with conventional note-taking methods (22). Thus, the cognitive benefits of mind mapping are not only theoretical but empirically supported, making it an ideal complement to OBE and hybrid pedagogical strategies.

The integration of mind mapping with hybrid learning represents an innovative approach to cognitive skill development in anesthesiology training, addressing the specific needs of complex medical knowledge retention. The high satisfaction rates among trainees strongly align with prior studies showing that a majority of students reported enhanced learning interest with mind mapping, while most students expressed satisfaction with mind mapping as their learning method (23). Our study extends these findings by combining mind mapping with online–offline hybrid teaching, creating a more comprehensive learning environment that enhances both engagement and knowledge retention.

These findings are consistent with broader evidence from active learning and flipped classroom interventions within residency and procedural specialty training. In anesthesiology, Wang et al. demonstrated that a flipped classroom model significantly improved residents’ knowledge test scores and self-efficacy compared with traditional didactics (24). Similarly, Liebert et al. found that surgical residents trained under a flipped model exhibited greater procedural confidence and skill retention (25). More recently, Zheng et al. conducted a meta-analysis showing that hybrid and active learning approaches in surgical education led to significant improvements in technical performance and learner satisfaction across procedural specialties (26). Collectively, these findings reinforce the effectiveness of integrating OBE with flipped and hybrid strategies, particularly in fields like anesthesiology and surgery where procedural competence and decision-making are essential outcomes. The improved practical competencies observed in our study correspond with meta-analysis findings showing that simulation-based education increased the overall success rate compared with traditional education with moderate certainty of evidence (27).

Furthermore, the enhanced self-directed learning ability observed among residents in this study aligns with the lifelong learning orientation that defines professional medical education. As Knowles’ framework suggests, adults are motivated by the relevance of learning to their work performance and career development. By allowing residents to identify learning needs, construct knowledge maps, and apply them during hybrid case-based discussions, the model operationalizes adult learning theory into measurable educational outcomes. This self-regulated process strengthens not only cognitive understanding but also metacognitive awareness—skills critical for sustained professional competence.

The unique contribution of our approach lies in its systematic integration of three pedagogical elements: OBE, hybrid learning, and mind mapping, addressing previous limitations in practical skill development. The enhanced self-directed learning abilities demonstrated by our study group reflect similar outcomes in previous research, where students achieved substantial improvements in Standardized Competence Tests, with performance improvements of more than 20 percentage points above national averages (28). Our study advances this finding by specifically tailoring the approach to anesthesiology resident training, demonstrating its effectiveness in specialized medical education. Teachers’ positive attitudes toward OBE, as previously documented through quantitative findings, support the feasibility of our integrated approach, despite the acknowledged implementation challenges (1). The successful implementation in our study provides a practical framework for overcoming commonly reported implementation challenges, offering concrete solutions for educational institutions.

This study has several limitations. First, the educational outcomes measured correspond to the lower levels of Kirkpatrick’s evaluation model—mainly knowledge acquisition and learner satisfaction—without assessing higher-level outcomes such as behavioral change, technical skill performance, or patient-related outcomes. Future research should include objective measures of clinical competence and long-term follow-up to evaluate transfer to workplace performance. Second, although randomization ensured comparability between groups, ethical considerations arise because residents in the control group did not initially experience the more interactive and effective OBE–hybrid–mind-mapping model. To address this, the same training package will be offered to control-group participants in subsequent program cycles to ensure equitable educational opportunities. Finally, the study was conducted in a single institution with a limited sample size, which may restrict generalizability. Multicenter studies with larger and more diverse populations are needed to validate these findings.

To conclude, the integration of OBE principles with online–offline hybrid teaching and mind mapping offers a comprehensive and effective educational framework for anesthesiology residency training. This model not only strengthens residents’ theoretical understanding and practical competencies but also cultivates self-directed learning and reflective thinking—attributes that are essential for lifelong professional growth. By promoting learner autonomy, critical reasoning, and the ability to integrate theory with practice, this approach aligns closely with the evolving needs of modern anesthesiology, where precision, rapid decision-making, and interdisciplinary collaboration are critical. Clinically, the model provides an innovative pathway to enhance procedural safety, improve communication within perioperative teams, and support evidence-based practice through better cognitive structuring of knowledge. From an educational standpoint, it demonstrates that thoughtfully designed hybrid strategies grounded in adult learning theory and cognitive principles can effectively bridge the gap between classroom learning and clinical performance. Future research should extend this framework to other procedural specialties, employ longitudinal evaluations to track behavioral and patient-related outcomes, and incorporate objective performance assessments to determine its impact on real-world clinical effectiveness. With further validation, this integrated teaching model holds promise for improving both the quality of residency education and, ultimately, patient care outcome.

## Data Availability

The raw data supporting the conclusions of this article will be made available by the authors, without undue reservation.
